# Navigating from a Depth Image Converted into Sound

**DOI:** 10.1155/2015/543492

**Published:** 2015-02-05

**Authors:** Chloé Stoll, Richard Palluel-Germain, Vincent Fristot, Denis Pellerin, David Alleysson, Christian Graff

**Affiliations:** ^1^University Grenoble Alpes, LPNC, 38000 Grenoble, France; ^2^CNRS, LPNC, 38000 Grenoble, France; ^3^University Grenoble Alpes, GIPSA-Lab, 38000 Grenoble, France

## Abstract

*Background.* Common manufactured depth sensors generate depth images that humans normally obtain from their eyes and hands. Various designs converting spatial data into sound have been recently proposed, speculating on their applicability as sensory substitution devices (SSDs). *Objective.* We tested such a design as a travel aid in a navigation task. *Methods.* Our portable device (MeloSee) converted 2D array of a depth image into melody in real-time. Distance from the sensor was translated into sound intensity, stereo-modulated laterally, and the pitch represented verticality. Twenty-one blindfolded young adults navigated along four different paths during two sessions separated by one-week interval. In some instances, a dual task required them to recognize a temporal pattern applied through a tactile vibrator while they navigated. *Results.* Participants learnt how to use the system on both new paths and on those they had already navigated from. Based on travel time and errors, performance improved from one week to the next. The dual task was achieved successfully, slightly affecting but not preventing effective navigation. *Conclusions.* The use of Kinect-type sensors to implement SSDs is promising, but it is restricted to indoor use and it is inefficient on too short range.

## 1. Introduction

Blindness and certain visual disabilities may be partially compensated by artificial retinae, that is, with light sensors directly implanted in a person's nervous system [1, 2]. However, less invasive sensory substitution devices (SSDs) may also be used allowing “the use of one human sense to receive information normally received by another sense” [3]. In this field, Bach-y-Rita [4] developed different kinds of devices to translate visual information into tactile feedback (essentially vibrations) felt by users' fingers, backs, or even tongues. The principle is often the same: a video camera extracts information from the user's environment, which is converted into vibrations felt by a specific body part.

In the present work, substitution is also initiated from optical data, but these are processed to deliver specific information on depth. In addition, the signal delivered to the user is auditory. This sensory modality has also been exploited before for navigation. Depth perception is a major source of information for navigation. For example, it is used to anticipate trajectories and avoid obstacles. In humans, depth perception is mainly visual. Beyond what we can touch, vision provides more qualitative and quantitative information than other senses. In the absence of visual input, especially in blind people, an additional signal can provide information on depth [5]. Thus, our novel system is based on translating an array of depth points into a mix of simultaneous sonic signals. Every point in the array potentially produces a pure sound (defined by intensity, tone, and stereo gain) and the melody of the overall sound is supposed to be representative of the visual scene. Such complexity of information may, or may not, be of advantage to the user. Before considering further adaptation for blind people, we decided to test its applicability to an elementary walking navigation task.

### 1.1. Visual-Auditory Substitution

Our system is inspired by auditory substitution devices that encode visual scenes from a video camera and produce sounds as an acoustic representation called a “soundscape.” In the vOICe (for Oh I See) [6], grey tones of a 64 × 64 pixel natural image are turned into sound through a left-to-right scanning of the visual scene. Learning this kind of image-to-sound conversion allows daily object localization and recognition [7], navigation (http://seeingwithsound.com/ website), or even letter recognition [8]. More impressively, after only 70 hours of training, congenitally blind people passed the blindness acuity test of the World Health Organization [9].

Loomis et al. [10, 11] also developed a Personal Guidance System to improve navigation without vision. They tested the efficiency of various visionless guidance techniques [2] and navigation abilities [11]. They found that virtual sound guidance had a clear advantage over spatial language on navigational performance, but only when the navigation task was combined with a dual task (i.e., a vibration detection task) [11–13]. The authors argued that virtual sounds can be immediately perceived and understood as spatial information, whereas spatial language requires that semantic information be processed and transformed into a spatial representation.

Other authors worked on navigation performance via an SSD called Brainport [3, 14]. Initially developed by Paul Bach-y-Rita, this device provides 2D information of the environment by electrotactile stimulation on the tongue. Recently, Kaiser [3] trained eight blind people to use the Brainport device. They compared navigation performances (travel time and contacts with walls) in a naturalistic environment before and after training program by using Brainport only, white cane only, and both. In the posttraining session, participants gained certain mobility: they were able to navigate in the courses with less contact than in the pretraining session. They were more precise in their navigation even if training did not lead to improving time travel.

Various systems differ in the way they convert images into sounds. As mentioned before, some techniques use the sequential scanning of columns in images. By processing only one column at a time [6], early visual-auditory SSDs successfully reduced 2D image information rate. On this basis, Cronly-Dillon et al. [15] translated columns into musical form after high-contrast line selection preprocessing.

More recent systems handle an entire 2D image snapshot in various ways. Taking the human vision system as a model, a multiresolution retina can encode the center in greater detail than in the rest of the field [16]. Horizontal encoding feeds the binaural intensity of stereo sound sources. For instance, González-Mora et al. [17] improved sound generation with the acquisition of “stereo pixels” depth map and spatialization techniques based on head-related transfer function (HRTF) for binaural encoding. In the VIBE [18], the “retina” is implemented with a reduced number of receptive fields (RFs), so that each of them can be assigned a specific sound source. Another system, called See ColOr (Seeing Colours with an Orchestra) [19] converts colors into different musical instrument sounds. It enhances colored object and texture perception and simultaneously converts depth into sound rhythm.

### 1.2. SSD Based on Depth Information

Only a limited number of audition-based SSDs focus on depth acquisition. Yet in vertebrates, different procedures evolved to evaluate object distance independent of vision and touch. For instance, echolocation was first discovered for bats in the air [20] and then for whales in the water [21]. The ultrasonic probing signals involved in these procedures are of an acoustic nature, both at the emission and the reception stages. Humans can also use acoustic signals containing spatial parameters to facilitate navigation and infer depth information from acoustic stimuli processing [22]. For instance, the Sonic Pathfinder is an electronic travel aid (ETA) that turns depth taken from an ultrasonic emitter-receiver system into sound [13]. However, depth is usually considered an optical-relevant feature, and man-made devices usually turn* optic* parameters into acoustical stimuli that are eventually processed by the brain to deduce depth. Interestingly, totally blind people are more efficient than sighted people at distance discrimination via auditory information [22].

More recently, the emergence of the RGB-D Kinect sensor has paved the way for a new generation of navigation systems by SSDs based on real-time depth map acquisition [23–28]. For example, Hicks et al. [24] developed an augmented reality system for partially sighted individuals, reducing the amount of information in the depth map. In this system, distance is converted to brightness and displayed on stereo 12 × 8 LED images mounted on ski goggles. In the same vein, Ribeiro et al. [27] retraced a sound image of an indoor environment with plane and face detection, generating low-pitch clicks when encountering obstacles. However, a long training period seems necessary to properly use the device.

**Figure 1:**
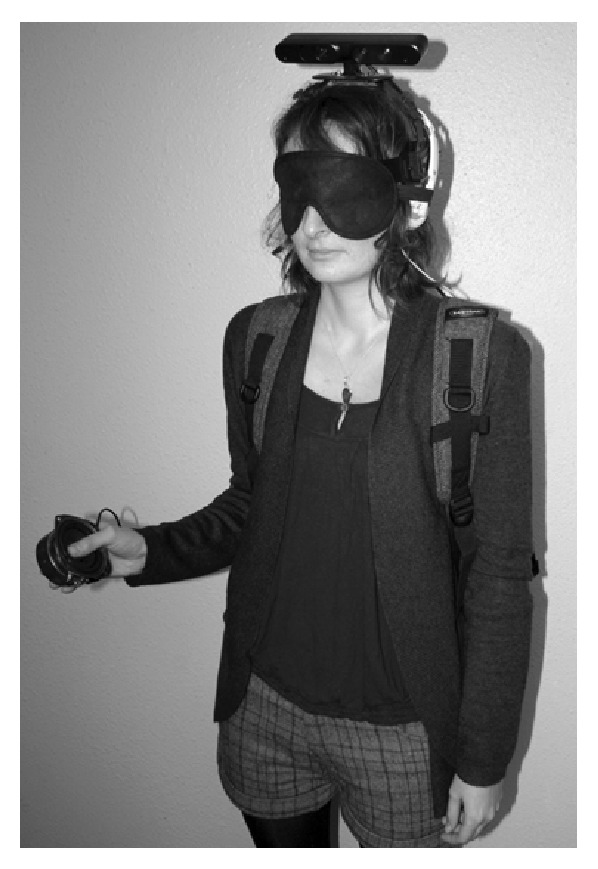
A blindfolded participant equipped with the set-up called MeloSee. The participant holds the distraction apparatus in her right hand.

We propose an SSD prototype constructed based on Kinect principles called MeloSee. The emitter-receiver system is mounted on the user's head (Figure 1), thus leaving his/her hands free for other tasks. It implements original methods to encode the depth environment so as to generate sounds from depth and azimuth [29]. We use a depth map provided by the RGB-D camera as an input. We resample the map using a limited number of receptive fields. The response associated with each receptive field is transcribed into sound. For the user's comfort and discrimination, we associate each RF's vertical position with a sound frequency on the musical scale. A differential stereo gain is also applied to the horizontal position. This generates a melody associated with the image in real-time. The development of a similar system was recently motivated by an inquiry among blind people [30].

### 1.3. Evaluation

Processing optical data to infer depth from the surrounding environment requires attention and cognitive resources. MeloSee readily extracts depth information in real-time and transposes it along acoustical scales. The user's resources may be spared and left available for tasks other than navigating.

The goal of our research is to test this depth-to-sound design in a navigation task. Firstly, does it at least allow travelling between walls without sight and without touch via only auditory clues? Secondly, if the depth-to-sound conversion is relevant, a small amount of training should be sufficient to achieve navigation, even in an unknown space. Thirdly, if learning is effective, it should remain for days without continuous refreshing (long-term learning). Finally, if the depth-to-sound comprehension is easy enough for users, the participants should be able to complete a distractive task while navigating. To test these four ideas, blindfolded participants navigated on different unknown paths for two sessions separated by a one-week interval. In each experimental session, they also participated in two trials with a distractive task. Travel time and errors (contacts with walls and U-turns) were recorded.

## 2. MeloSee Specifics

**Figure 2:**
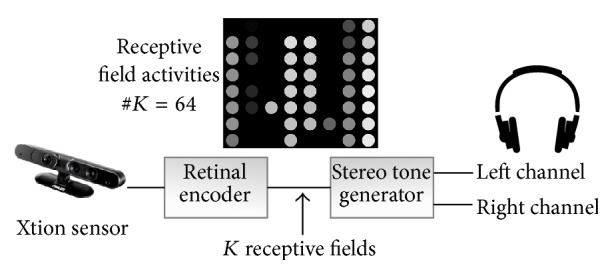
Visual-auditory sensory substitution flowchart. The high-input information throughput is significantly reduced before being converted into sound.

The system presented in Figure 2 comprises two main components: a retinal encoder which extracts only relevant information from the image and a sound generator which produces a stereophonic melody. An RGB-Depth ASUS Xtion camera (updated version of Kinect sensor), mounted on participants' heads, supplies the depth map as input to the retinal encoder, and the sound is delivered real-time to them through headphones. More precisions about the device are given elsewhere [29].

### 2.1. Retinal Encoder

The Asus Xtion sensor projects an infrared pattern on the scene. (For this reason, the system does not work properly outdoors.) Depth can be inferred from the deformation of the infrared pattern [31]. The depth sensor supplies a VGA (640 × 480 pixel resolution) video stream of the scene, coded on 11 bits at 30 Hz. We introduced RFs to subsample the input images selectively. As mentioned by Fristot et al. [29, page 1991] “As the entire visual information of the image (all the pixels) cannot be converted simultaneously into sound without making a cacophony, compression of visual information is needed.”

**Figure 3:**
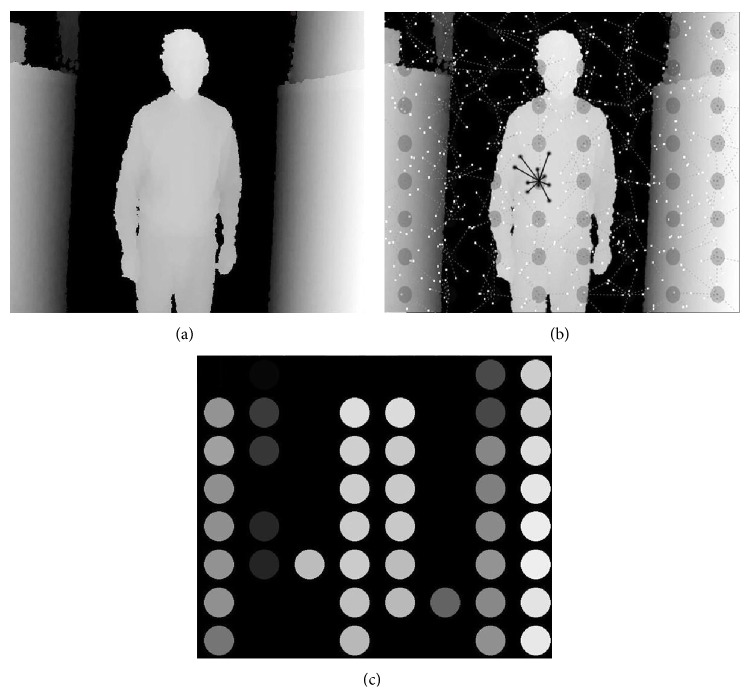
Retinal depth encoder. (a) Grayscale depth map. (b) Activity computation. (c) RF activities: the closer the object, the lighter the disc (Figure 3(b)) and the louder the sound; the farther the object, the darker the disc and the softer the sound.

An example of this approach is shown in Figure 3 for a grayscale depth map. A regular grid of 64 RFs covers the overall image. Each RF extracts depth from a fixed group of 10 neighboring pixels around its center. To prevent from aliasing, pixels are sampled randomly from a 2D normal distribution [29]. For an illustration of the material and the experimental set-up, see it in the Supplementary Material available online at http://dx.doi.org/10.1155/2015/543492.

### 2.2. Stereo Tone Generator

Each RF's activity is transformed into a particular sound source. Pitch is then associated with each RF according to its vertical position (from C_4_ to C_5_ on an octave scale, with low frequencies at the bottom and high frequencies at the top). The horizontal position defines stereophonic left and right gain (for amplification or attenuation) applied to the source of the binaural sound representation. Intensity is translated inversely proportional to distance. Sounds for all RFs are played in parallel. A stereo tone generator outputs the auditory scene by adding the contributions of all RFs as a synthesizer with linear contributions from oscillators. The audio update rate was 7.5 Hz or 132 ms.

The Asus Xtion sensor cannot operate closer than 50 cm from the targeted object or surface. Below this minimum distance, the set-up becomes silent. Sound intensity also fades with distance, down to zero at a certain maximum limit. For our experiment, the maximum was adjusted to 250 cm. While we could set the sensor to operate farther away, doing so would reduce the sound intensity contrast at closer distances.

### 2.3. Time Latency

In a sensory-motor task, time latency is a key feature for the system. We accurately assessed the latency of whole images to sound conversion processing using a blinking LED, shot by the RGB-D sensor, which also triggered an oscilloscope (because the LED light prevents depth calculation at the dazzled point). Based on this, time latency is the difference between the LED extinction and the beep's arrival. With a midrange laptop (Win7, Asus PC Intel Atom N550 CPU), we measured a latency of approximately 100–150 ms, which is commendable for a real-time system.

## 3. Behavioral Assessment

### 3.1. Participants

Twenty-one healthy participants took part in the study (14 women; Age: Mean = 21.2, Standard deviation = 2.1). They were students at Grenoble University. They were paid for their participation in each session.

The study was conducted in accordance with the Helsinki Declaration and with the understanding and a written consent of each participant.

### 3.2. Procedure

#### 3.2.1. Navigation Task

Participants were blindfolded with a sleep eye mask during the entire experiment. Therefore they never saw the experimental set-up. Then, they were led to a starting point and using MeloSee they were asked to walk until the end of a path. They were asked to navigate as rapidly and accurately as possible, that is, making the least possible contact with walls and screens. Before starting the trials, they were informed that there were no barriers, stairs, cul-de-sacs, or corridors narrower than a doorway along the route. As participants had to do not touch the walls, the task could only be performed with SSD switched-on, and so no baseline trial was recorded (e.g., blindfolded participants walking with switched-off SSD). During some trials they had to complete a concurrent discrimination task monopolizing both hands while navigating. Therefore, there was no comparative trial with another device requiring the hand (e.g., a control group using a white cane). Importantly, during the trials, participants were warned when they started going in the wrong direction (i.e., U-turns).

**Figure 4:**
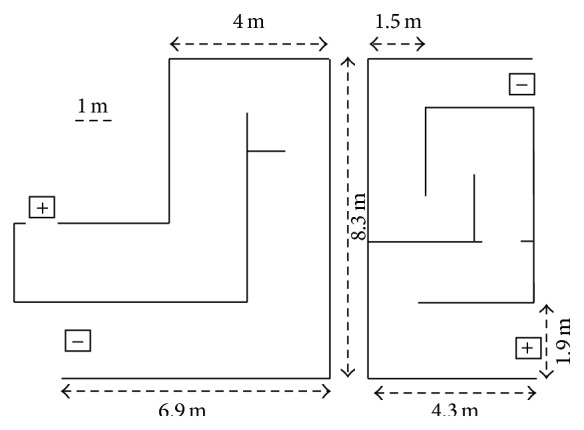
Paths used in the experiment. Path A (left) was 22 m long and path B (right) was 19.6 m long. Squares indicate either the start or finish, depending on the route direction.

Two different paths (Figure 4) were built inside large rooms, using walls and large paper sheets that covered standing poster grids in a continuous screen, from one to two and a half meters above the ground. Path A (22 m) was slightly longer but path B (19.6 m) was narrower and had more corners. Switching the direction (from start to finish) for each path yielded a total of four different routes: A+ and A−, B+ and B−. The four different routes were repeatedly navigated by each participant in a series totaling 12 trials. Performances in the four series were grouped and analyzed together.

#### 3.2.2. Distraction Task

The distraction task required participants' attention without impeding “melody” hearing. It consisted of detecting a temporal pattern of touches. The participant's thumb was in contact with a bare loudspeaker held in one hand and connected to an MP3 player. The touch stimuli consisted of 100 Hz sinus-wave buzzes lasting for 200 ms each. They were not audible by participants during the navigation task but strong enough to stimulate their skin. Buzzes were emitted alone, by pair, or by triplet, with a one second interbuzz onset interval. The patterns themselves were separated by random intervals of 2.7 to 12.0 s and assembled in blocks lasting for 30 s. Each block contained one pair, one triplet, and two single-buzz patterns, all randomly distributed. Ten blocks were assembled in a 5 min audio file played in a loop. While navigating, participants had to respond to double buzzes, and only double buzzes, by clapping their thigh with their free hand. Their performance was monitored and assessed in real-time with the help of a waveform track representing the sequence of buzzes. Because the study focused on navigation performance, participants were asked to prioritize the distraction task each time it was a part of a trial. Thus, defining navigation performance as secondary for all participants prevented various trade-offs between tasks that would obscure the actual distracting effect expected for navigation.

### 3.3. Design

#### 3.3.1. Familiarization

Prior to the first trial of the first session, participants were familiarized with the upcoming distraction task and the SSD in an anteroom. First, they were told to recognize the double buzz within a 30-second sequence. Then, the rationale of the SSD system was explained to them while they were equipped and blindfolded. The way sound intensity changed with distance from walls or screens was demonstrated (and experienced) in a didactic exchange with the experimenter. Emphasis was placed on the importance of head movements. Additional explanations were given on the system becoming silent at extreme-short range and when facing wide, open spaces devoid of obstacles. Then, participants moved around for two minutes in the anteroom in order to understand the sound coding. During this task, they were allowed to use their hands to explore the room with both touch and sound. Finally, they experienced the distraction task, together with the substitution system, for one minute. The overall familiarization lasts less than eight minutes and all participants felt ready for the navigation task.

#### 3.3.2. Experimental Sessions

Two sessions, each lasting less than one hour, were separated by one week (intersession interval M = 7, SD = 2 days). Each session included six trials.

The experimental design was within-participant, and the general procedure was the same in each session. Each participant was randomly assigned series # 1, 2, 3, or 4 (Table 1) in pseudorandom order. Participants navigated the same first route three times (1_1_, 1_2_, and 1_3_). They then navigated a second route (2_1_). This was done to test for short-term learning independently of route-learning. Finally, they ran again the second and first routes (**2**_**2**_ and** 1**_**4**_) while performing the distraction task. Before each trial, participants were informed whether the route (not the* path*) to be navigated was new or had already been run in a previous trial. This was done to prevent guessing that would inject variability among participants.

The same procedure was applied the second week to test for long-term learning. Inverting travel direction for the two paths resulted in two new routes for the participants.

### 3.4. Data Analysis

As previously noted, participants had to navigate as quickly as possible without touching the walls. For each trial, we measured travel time and navigation errors (number of contacts with the walls and U-turns). The performance of the distractive task was also recorded. For each dependent variable, a repeated-measure ANOVA was conducted with week session (first, second) and trial ranks (1_1_, 1_2_, 1_3_, 2_1_,** 2**_**2**_, and** 1**_**4**_) as independent variables.

## 4. Results

### 4.1. Travel Time

**Figure 5:**
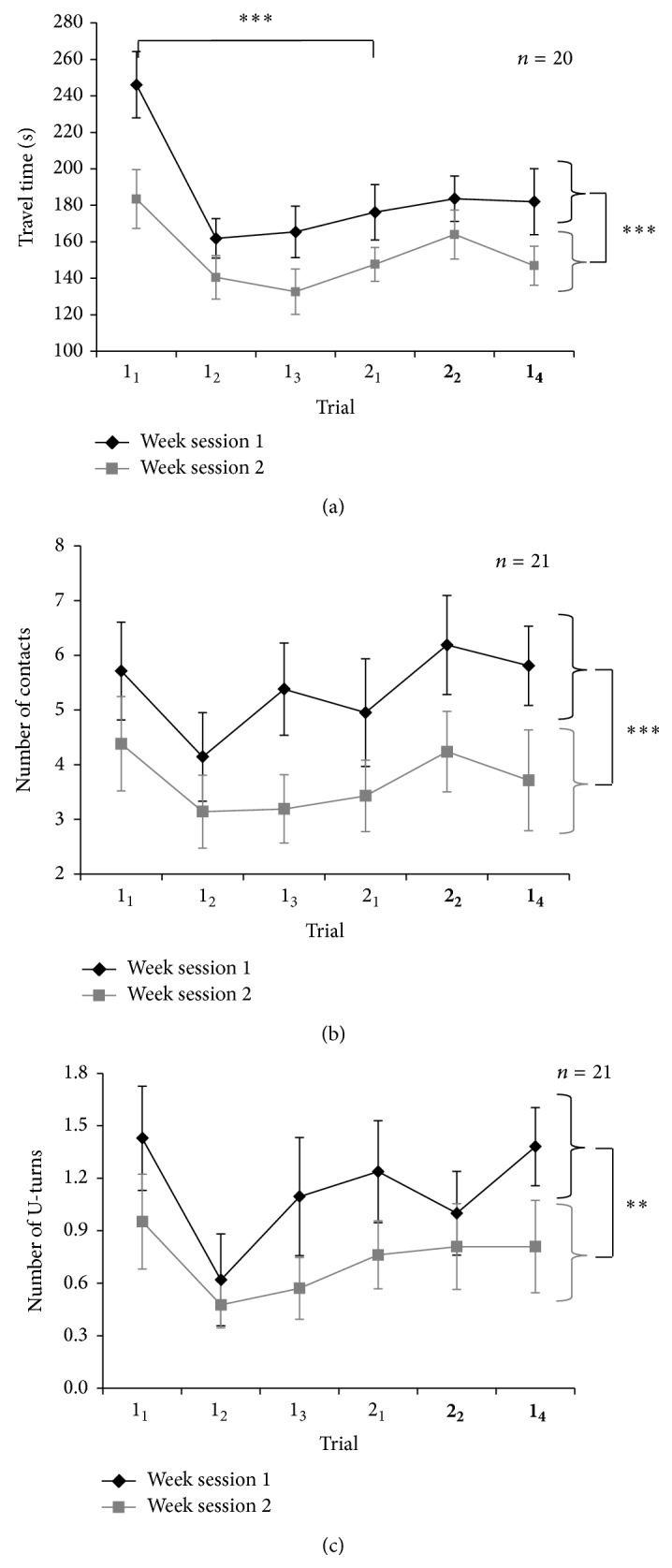
Navigation performance in the six experimental trials during the first and the second week sessions. Trials 1_1_, 1_2_, 1_3_, and 1_4_ are conducted on the same path and trials 2_1_ and 2_2_ on another one. Cognitive load was added in trials **2**_**2**_ and** 1**_**4**_. Top panel (a): travel time in seconds (note that the *Y*-axis starts from 100 s); middle panel (b): number of contacts with the walls; bottom panel (c): number of U-turns. Error bars represent standard errors of the means. Stars indicate significant differences with ^**^ *P* < 0.01; ^***^ *P* < 0.001.

The results for travel time are presented in Figure 5(a). No interaction effect was found (*P* > 0.5) between week session and rank trial, which suggests that the performances from trial to trial were similar in the first and in the second experimental session. Interestingly, a main effect of trial rank, F(5, 95) = 13.26, *P* < 0.001, indicated that navigation performance changed within experimental sessions. A main effect of the week session was also observed, F(1, 19) = 22.12, *P* < 0.001, showing an improvement in navigation performance from the first to the second experimental session (long-term learning). The simple-effect analysis of trial rank showed a significant difference between trial 1_1_ and trial 2_1_ (both novel paths), F(1, 19) = 30.05, *P* < 0.001. Thus, the first trial of the second path (trial 2_1_) was accomplished faster than the first trial of the first path (trial 1_1_) suggesting short-term learning independent of path familiarization. No significant difference was found between the last trial of the first path (trial 1_3_) and the first trial of the second path (trial 2_1_). To illustrate this long-term improvement, we compared mean navigation velocity to complete the first trial of the second path (2_1_). Participants navigated approximately 7 m/min the first week against 8.7 m/min the second one.

When a cognitive load was added, travel time increased for the second route (trial** 2**_**2**_) only F(1, 19) = 4.81, *P* < 0.05. Furthermore, no significant difference was found between trial 1_3_ and trial** 1**_**4**_ (*P* > 0.05). The cognitive load had no significant impact on travel time for the route already navigated three times before. It is worthy to note that the distraction task was correctly completed (day 1, trial 1: 87%, SD = 10; day 1, trial 2: 80%, SD = 15; day 2, trial 1: 90%, SD = 14; day 2, trial 2: 84%, SD = 12).

### 4.2. Navigation Errors

An ANOVA on contact with the walls (Figure 5(b)) showed no significant interaction between session and trial rank (*P* > 0.05) and no significant trial rank effect (*P* > 0.05). The analysis showed a main effect of the week session, F(1, 20) = 37.09, *P* < 0.001. Participants made significantly less contact in the second week session than in the first one.

The same pattern of results was observed with the number of U-turns (Figure 5(c)). There was no interaction between session and trial rank (all *P*s > 0.05). The ANOVA showed a main effect of the week session F(1, 20) = 14.79, *P* < 0.01. Participants made significantly fewer U-turns in the second than in the first session. Distraction task showed no significant effect on contacts (*P* > 0.5) or U-turns (*P* > 0.5). A significant correlation was observed between the number of contacts and U-turns (*r* = 0.74, *P* < 0.05). Analysis conducted with combined data yielded the same results as those on separated error data presented here.

## 5. Discussion

Together, our results seem to show the applicability of depth image conversion into sound for navigation along unknown paths. Interestingly, we noticed time improvement between a first and a second trial performed in the same path. We also observed time improvement between the first trial in the first path and the first trial performed in a new path (short-term learning). Performance also improved over sessions (long-term learning), even when a distraction task was introduced.

It is important to consider that the aim of this study was to test the system alone in a basic navigation task. The experimental procedure was operational enough to assess performance with different quantitative variables and with an additional cognitive load that may be further adjusted in complexity. The paradigm may inspire assays for future development of the same real-time device or to compare different SSDs later.

Our system was employed readily by inexperienced users (with only eight minutes of familiarization with the device). Travel time decreased between the first and the second trials and remained lower along the remainder of each session. With even shorter travel times a week later, learning was also demonstrated to be of longer term. In addition to travel time, a decrease in navigation error frequency confirmed the long-term improvement, with the agreement of two additional measures. Moreover, when participants had to deal with a distraction task via tactile stimulation, learning was robust as evaluated by both travel time and errors. The distraction task itself was hardly affected in the process. The additional load somewhat lengthened travel time in the less-navigated route only. The effect was slight in comparison with the massive learning progress observed.

However, the speed performance, around 8.7 m/min at the best, may seem low in general as compared to other systems (e.g., [25]).

Our results seem consistent with other studies [12, 18, 24] showing that sound can profitably convey information to navigate in an unknown environment, using either 2D light reflection (as with vision) or depth (as with echolocation) as a primary input. For instance, in the Sonic Pathfinder [32], depth was evaluated from three-directional ultrasonic beams and turned into a monodic stereophonic sound stimulus. Our RGB-D system also delivers depth information directly, similar to such ETA. However, it provides a 2D array and a wide scope, compared to the restricted beam focus of the cetacean, bat, and pathfinder systems. Auvray [33] considers that an efficient SSD shares common features with natural sensory-motor coupling. Vision, which is normally used in navigation, projects a 2D light image from the retina to specific areas of the central nervous system. Interestingly, our system produces a depth image which projects a 2D sound image that may match such brain structures dedicated to space perception. Thus, the motor-vision coupling involved in navigation might be favored by our system's real-time operation. Moreover, rapid adjustment is crucial for moving around without vision. In comparison, the vOICe offers better substitution of vision for recognizing shapes like letters on a plane, but it is hardly informative for depth and may be too slow to allow online navigation.

Our system presents the advantage of being constructed with the RGB-D sensor, a common manufactured component. However, it has two functional limitations. First, because it is based on infrared beams, it cannot operate outdoors where it is jammed by stronger concurrent signals from sunlight. Other sensor systems could be considered as input devices for a version that would deliver a similar polyphonic signal from a depth array that could be sampled outdoors. Second, the RGB-D sensor we tested does not pick up information at very close range (0.5 m). Some participants' main difficulty was finding their way in narrow passes and confined places, such as corners or door frames. In this particular situation, they learned to step back to restore the acoustic signal, after which they succeeded in walking faster and better. In subsequent versions of the system, various methods have to be implemented to help participants discern between blanks at very close and very long range. Automated processing may additionally be implemented to help the user detect relevant patterns [32].

## 6. Conclusion

The goal of the present experiment was to show that our portable real-time SSD that turns a depth scene from RGB-D into polyphonic stimulation can be adapted into a usable SSD.

As shown by our test, using different paths, polyphonic conversion of a 2D depth array can help navigation in corridors without vision and without touch. Practice significantly improved through long- and short-term learning in both new and more familiar paths. The portable system remained functional even when a supplementary task diverted participants' attention. The quick online coupling between real space and auditory mapping seemed to connect with cognitive processing that is normally implemented from visual natural input. Its development could help sightless people find their way in unknown as well as more familiar paths, without monopolizing the navigator's hand and attention. Future works are however required to test the relative efficiency of our device compared to other SSDs or other guidance systems (white cane or dog) and to test our device with blind persons.

## Supplementary Material

This video is a live camera view from a participant walking the maze with the Sensory Substitution Device (SSD). MeloSee SSD converts depth in sound intensity, with stereo modulation for the horizontal axis. Height is converted in pitch (vertical axis). The left view corresponds to depth map with color encoding, from red for the closest (50 cm) to blue for the furthest distance (250 cm). Areas in black are either too far or too close to be detected by the Kinect® sensor and white areas are shade sensor artifacts. Note that black and white are not auditory encoded. The right view corresponds to RGB normal camera-view of the maze. The maze segment displayed here starts between two parallel screens, then turns 90° left after facing a wall.

## Figures and Tables

**Table 1:** Experimental block design for the two sessions.

Trial		Week session 1						Week session 2					
		1_1_	1_2_	1_3_	2_1_	2_2_	1_4_	1_1_	1_2_	1_3_	2_1_	2_2_	1_4_
Series	#1	A+	A+	A+	B+	**B+**	**A+**	A−	A−	A−	B−	**B−**	**A−**
	#2	A−	A−	A−	B−	**B−**	**A−**	A+	A+	A+	B+	**B+**	**A+**
	#3	B+	B+	B+	A+	**A+**	**B+**	B−	B−	B−	A−	**A−**	**B−**
	#4	B−	B−	B−	A−	**A−**	**B−**	B+	B+	B+	A+	**A+**	**B+**

Each series (#1 to #4) started with a different route and used the four routes (A+ and A−, B+ and B−) in a different order. In each session, the second run of the second route (2_2_) and the fourth run of the first route (1_4_) were navigated while performing a distractive task (bold routes). For each participant, same-order trials between the two sessions were run on the same path (either A or B) but in the other direction (either + or −).
